# Decoupled Graph Attention Modeling and Anomaly Traceability Method for Multisystem Coupling in SLM Equipment

**DOI:** 10.3390/s26123889

**Published:** 2026-06-18

**Authors:** Qi Liu, Weijun Liu, Hongyou Bian, Fei Xing

**Affiliations:** 1 School of Mechanical Engineering, Shenyang University of Technology, Shenyang 110870, China; 2 Nanjing Zhongke Raycham Laser Technology Co., Ltd., Nanjing 210046, China; xingfei@raycham.com; 3 School of Mechanical Engineering and Automation, Northeastern University, Shenyang 110819, China

**Keywords:** selective laser melting, predictive health management, multisystem coupling, graph attention network, anomaly traceability, decoupled representation

## Abstract

Selective laser melting (SLM) equipment operates as a complex cyber–physical system, wherein strong implicit coupling among internal subsystems presents significant challenges for condition monitoring and fault diagnosis. Existing deep learning methods often suffer from feature submersion when processing multi-source heterogeneous data and lack the capability for system-level topological causal inference. To address these issues, we propose a multisystem coupling modeling and anomaly traceability method based on a decoupled graph attention network (ST-DBGAE). Independent local spatiotemporal feature alignment modules are constructed to map heterogeneous sensory data into a unified latent space. This eliminates dimensional discrepancies while strictly maintaining the feature independence of underlying hardware subsystems, such as optical and gas circuits. A dynamic graph attention mechanism with sparse priors is subsequently introduced to adaptively capture time-varying coupling weights triggered by implicit interactions (e.g., thermal fluids), bypassing the need for predefined rigid physical connections. Furthermore, a dual-branch two-stage decoupled optimization architecture is designed. By blocking the cross-interference of global backpropagation, this architecture outputs a continuous equipment health index (HI) based on reconstruction errors and employs a topological difference matrix inference mechanism to reversely anchor the root-cause nodes responsible for cross-system cascading degradation. Experimental results based on over 310,000 real operational monitoring records from industrial SLM equipment demonstrate that the proposed model achieves a comprehensive diagnostic Macro-F1 score of 96.5% across eight operating states. The single-class detection rates (ACCs) of specific underlying anomalies are significantly improved. This method not only enables high-precision equipment health warnings but also provides a physically interpretable microscopic fault propagation mapping for predictive maintenance.

## 1. Introduction

Selective laser melting (SLM) serves as a core technology in metal additive manufacturing. Its long-term operational reliability directly dictates its viability for high-end engineering applications [1]. Industrial SLM equipment features prolonged single-forming cycles and substantial hardware costs. Under continuous full-load operating conditions, component aging, environmental drift, and operational disturbances frequently trigger unplanned downtime [2]. Severe equipment-level faults not only cause part rejection but can also inflict irreversible damage on core components, such as high-power lasers and precision galvanometers. Therefore, transitioning from reactive maintenance to an equipment-level condition monitoring and predictive health management (PHM) system is a critical prerequisite for ensuring the stability of the additive manufacturing process [3].

**Figure 1:**
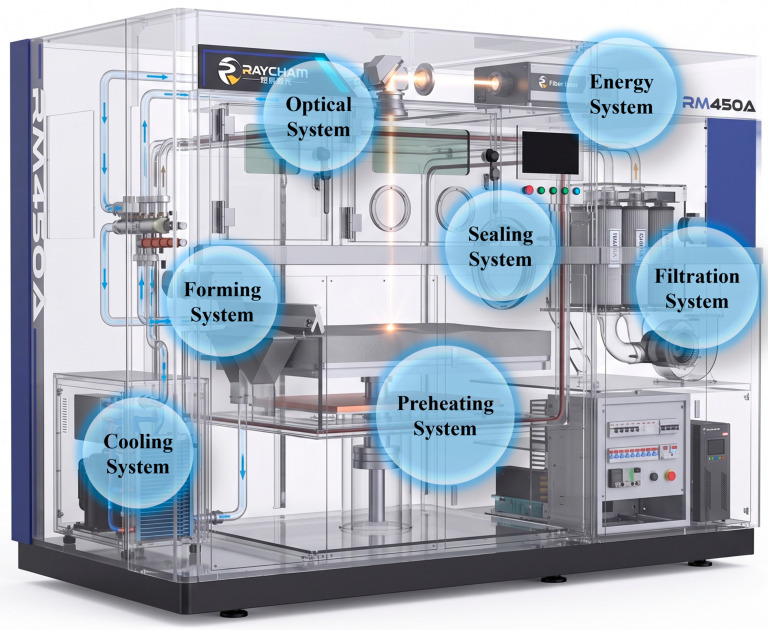
Multisystem architecture of SLM equipment.

Distinct from traditional numerical control machining equipment, SLM systems exhibit typical cyber–physical system (CPS) characteristics. Their overall operation relies on the strict spatiotemporal coordination of heterogeneous subsystems, including optical, cooling, forming, and sealing gas circuits. As illustrated in Figure 1, this multisystem architecture exhibits pronounced coupling sensitivity. Local disturbances within a single subsystem easily cross physical boundaries, triggering cascading degradation through implicit interaction links like thermal and fluid dynamics. Taking the gas circuit system as an example, minor fluctuations in filter pressure drop disrupt the protective gas flow field within the processing chamber. The resulting flow turbulence hinders aerosol clearance, which in turn induces a thermal lensing effect on the laser protective lens, ultimately causing forming energy attenuation and overall operational deterioration. These degradation mechanisms indicate that anomalous evolution in SLM equipment manifests as complex topological propagation. Consequently, isolated monitoring based on a single sensory variable struggles to accurately characterize the global health evolution trajectory.

Condition monitoring based on multi-source sensor networks is fundamental for ensuring system reliability. The application of deep learning has driven the fault diagnosis paradigm from feature engineering to data-driven approaches. In additive manufacturing, Chen et al. [4] and Petrich et al. [5] demonstrated that multi-sensor fusion effectively overcomes the blind spots of single sensory information. However, directly applying existing data-driven paradigms (e.g., 1D-CNN or LSTM) to SLM equipment with “multi-system strong coupling” characteristics faces severe limitations. The global mapping mechanism inherently restricts root-cause traceability. Gurav et al. [6] pointed out that while such black-box end-to-end models yield high classification accuracy, they lack fine-grained structural anomaly representations. When a warning is triggered, the model cannot reverse-parse the underlying independent hardware causing the global degradation.

Furthermore, multi-source monitoring data exhibit significant heterogeneity, manifesting as mismatches in sampling frequencies and discrepancies in physical dimensions. For temporal alignment, Xie et al. [7] identified temporal and dimensional mismatches as core barriers. Simsir et al. [8] and Bevans et al. [9] emphasized that extracting synchronous evolution features is foundational for capturing dynamic degradation rules. Regarding spatial feature mapping, traditional early fusion (concatenation) strategies carry a severe risk of “feature submersion”. Sindhu et al. [10] and related research [11] revealed that macroscopic environmental variables with high numerical variances dominate global gradients, masking critical early electromechanical degradation precursors. To mitigate dimensional conflicts, architectures like multimodal variational autoencoders [12,13] map data into a globally shared latent space. Although mitigating absolute scale differences, the low structural transparency of such latent spaces restricts engineering interpretability [14]. As Wang et al. [15] noted, pursuing global diagnostic accuracy by blending independent subsystem states fundamentally destroys original hardware physical topology boundaries, yielding spurious correlations rather than real physical transmission links.

Resolving this requires explicit topological modeling of the implicit physical coupling. Subsystem coordination mostly relies on thermal or fluid dynamics without explicit mechanical connections. Gao et al. [16] proposed a recurrent graph network embedded with physical priors, proving that explicitly constructing sensor influence graphs improves generalization. Graph Neural Networks (GNNs) abstract hardware as nodes and interaction relationships as edges. Liu et al. [17] and Xing et al. [18] confirmed that GNN frameworks effectively handle physical interactions, improving diagnostic robustness. However, mainstream Graph Convolutional Networks (GCNs) exhibit limitations for equipment dependent on implicit physical fields, as their feature aggregation relies on static adjacency matrices. Ma et al. [19] stated that static prior topologies struggle to represent complex time-varying relationships. In SLM, coupling strength exhibits dynamic characteristics that drift with operating conditions. Applying static graphs misses dynamic anomalous associations and leads to the over-smoothing of node features.

To break static topology limitations, graph attention networks (GATs) introduce adaptive mechanisms to learn implicit similarities, dynamically quantifying dependency weights [20]. Wen et al. [21] and Wang et al. [22] verified that attention coefficients effectively reveal dynamic coupling and prompt the network to automatically focus on core nodes triggering degradation. Recently, graph attention mechanisms have been successfully explored in additive manufacturing for micro-level defect prediction based on the geometric spatial proximity of melt pools [23]. However, applying such architectures to macroscopic equipment health monitoring based on 1D sensory data requires distinct theoretical justification. Unlike geometric spatial reasoning in melt-pool dynamics, equipment-level monitoring must map non-Euclidean functional topologies across physically isolated hardware boundaries. Applying GAT to 1D signals specifically addresses the risk of “feature submersion” inherent in macroscopic variables, shifting the graph paradigm from morphological defect detection to dynamic cross-system fault traceability.

To address these macroscopic challenges, this paper proposes a multisystem coupling modeling and anomaly traceability method based on a decoupled graph attention network (ST-DBGAE). The comparative novelty and main contributions of this study are structurally summarized in three dimensions:

1. Overcoming Entangled Representation via Decoupled Independent Alignment: Compared to traditional early fusion or global autoencoders that suffer from “feature submersion,” we design parallel local autoencoders. This structurally addresses dimensional conflicts and strictly maintains the feature independence of core hardware nodes, preserving weak degradation precursors before topological aggregation with-out global gradient cross-interference.
2. Breaking Static Topology Limitations via Sparse-Prior Dynamic GAT: Compared to traditional GCNs that rely on predefined, rigid adjacency matrices, we introduce a dynamic GAT with sparse prior constraints. This data-driven approach adaptively quantifies non-linear, time-varying implicit coupling weights (e.g., thermal and fluid interactions) while suppressing pseudo-correlation calculations, ensuring robustness under operating condition drifts.
3. Enabling White-Box Causal Inference via Two-Stage Optimization: Compared to standard black-box classification models, we implement a two-stage decoupled optimization to block joint backpropagation. This isolation enables the output of continuous equipment health indices (HIs) alongside a novel topological difference matrix ΔC algorithm, which reversely and precisely locates the source hardware and propagation links triggering global degradation, shifting the PHM paradigm from state alerting to structurally interpretable anomaly traceability.

## 2. Proposed ST-DBGAE Methodology

Before detailing the mathematical formulations of the ST-DBGAE, it is essential to establish the conceptual mapping between the physical SLM equipment and the proposed AI architecture. In this study, the physically isolated hardware subsystems (e.g., optical, cooling, and filtration systems) are strictly abstracted as independent graph nodes (V). Their implicit, non-Euclidean interactions—driven by thermal radiation and aerodynamic flow fields—are modeled as dynamic graph edges. To prevent the critical “feature submersion” of weak optoelectronic signals by macroscopic fluid variables with large variances, the methodology is designed following a strictly decoupled paradigm. As introduced below, the architecture first forces independent local feature alignment (stage one) before executing any global topological aggregation (stage two).

### 2.1. Overall Architecture Design and Problem Definition

**Figure 2:**
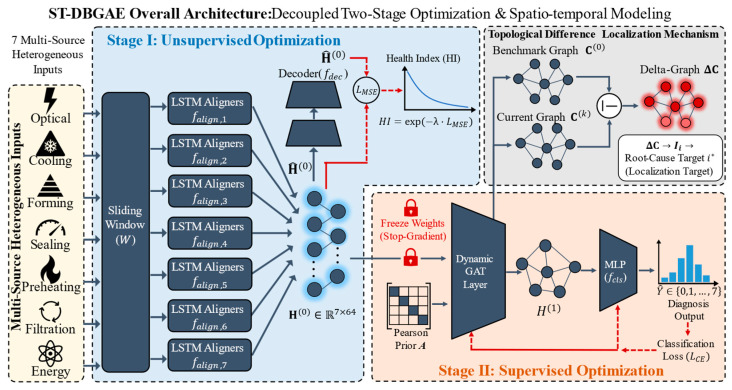
Overall architecture of the spatiotemporal dual-branch decoupled graph autoencoder.

The operation of SLM equipment is a multi-physics cross-medium coupling process. Under these conditions, cascading degradation manifests fundamentally as the non-linear distortion of implicit physical interaction rules and topological structures among systems [24]. To overcome the entangled representation limitations of traditional fusion models. We propose a Spatiotemporal Dual-Branch Graph Autoencoder (ST-DBGAE) based on two-stage decoupled optimization. It is crucial to clarify that in the context of this study, the term “spatial” does not refer to the Euclidean geometric distance between physical sensors. Instead, it strictly denotes the non-Euclidean topological space representing the functional coupling network across physically isolated hardware subsystems. The overall architecture is illustrated in Figure 2.

Mathematically, given multi-source heterogeneous monitoring data, we construct an input feature tensor $X\in R^{N\times W\times D}$ using a sliding time window. Here, N=7 represents the core hardware subsystems, W is the time window length, and D is the aligned feature dimension. The model outputs a continuous equipment-level health index (HI) and maps it to a discrete state label Y∈{0, 1, …, 7}. Label Y=0 denotes a healthy state, while Y∈{1, …, 7} denotes initial anomalous states of different subsystems.

To maintain physical topological boundaries, the model abandons the traditional end-to-end paradigm [25] in favor of four decoupled modules. The local spatiotemporal feature alignment layer employs independent channels to perform dimensional alignment on the N subsystems, generating initial representations with clear boundaries. The unsupervised state reconstruction branch utilizes an autoencoder to perform latent space reconstruction on the aligned features during stage one optimization, minimizing reconstruction loss to extract pure state representations and calculate a continuous HI curve. The dynamic graph attention core module, incorporating sparse priors [26], adaptively learns time-evolving implicit interaction weights among nodes. Finally, the supervised anomaly traceability branch utilizes cross-entropy loss during stage two optimization to classify discrete state labels based on global graph topology aggregation, reversely locating root-cause subsystems via offset difference analysis.

### 2.2. Feature Alignment and Implicit Solidification

Before constructing a system-level multi-field coupling graph, the underlying feature mapping barrier must be resolved. The 41-dimensional monitoring signals across the seven subsystems exhibit significant disparities in sampling resolution and physical dimensions. Direct global feature concatenation inevitably leads to feature submersion and the loss of physical boundaries [27]. Therefore, an independent spatiotemporal feature alignment mechanism is designed during stage one preprocessing.

Using a fixed-step sliding time window, the input feature matrix for the i-th subsystem is defined as $X_{i}\in R^{W\times D_{i}}$, satisfying $\sum_{i=1}^{N}D_{i}=41$. To address non-linear degradation characteristics, mutually independent LSTM networks serve as local temporal aligners $f_{align,i}$ [28]. Processing the W time series, the network extracts the hidden state of the final time step as the static comprehensive representation:

$$h_{i}^{\left(0\right)}=f_{align,i}\left(X_{i}\right)\left[-1\right]$$

where $h_{i}^{\left(0\right)}\in R^{d}$ (d=64 in this study). The N isomorphic hidden vectors are stacked along the node dimension to generate the initial graph node feature tensor

$$H^{\left(0\right)}=\mathrm{Stack}\left(h_{1}^{\left(0\right)},h_{2}^{\left(0\right)},\ldots ,h_{N}^{\left(0\right)}\right)\in R^{N\times d}.$$

This operation projects heterogeneous $D_{i}$-dimensional quantities into a unified d-dimensional space, eliminating dimensional differences while ensuring feature decoupling [29]. Furthermore, it solidifies the dynamic degradation trend within time window W into an aligned static tensor $H^{\left(0\right)}$, avoiding gradient calculation problems inherent in traditional sequence networks.

### 2.3. Dynamic Graph Topology Construction Based on Sparse Priors

**Figure 3:**
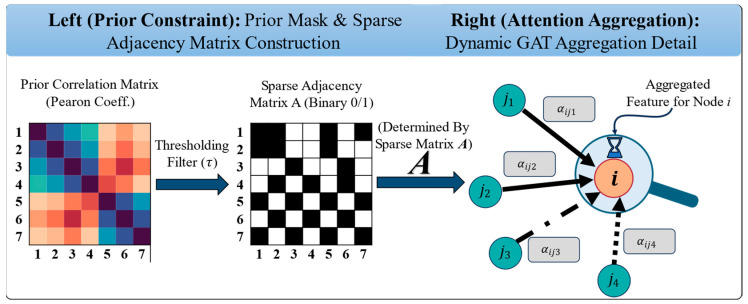
Dynamic graph topology construction and attention aggregation mechanism based on sparse priors.

After stage one converges and freezes the feature extraction weights of $f_{align,i}$, the model enters stage two topology inference. Assuming a complete graph topology in complex electromechanical systems violates underlying thermal and fluid constraints and introduces high-variance background noise. Using dynamic priors to construct correlation boundaries is vital to prevent over-smoothing [30]. Thus, the mutual information of multi-source data under normal operating states acts as the sparse prior constraint (Figure 3).

We calculate the Pearson correlation coefficient matrix $\rho \in R^{N\times N}$ among the N hidden-layer nodes using normal state samples [31]. A correlation threshold τ filters out weak random disturbances, generating a sparse adjacency matrix A.

$$A_{ij}=\left\{\begin{array}{cc} 1, & if|\rho_{ij}|>\tau \;and\;i\neq j\\ 0, & otherwise \end{array}\right.$$

This establishes a directed edge set E for system-level interactions.

Based on this sparse boundary, a GAT performs topological message passing. Addressing the dynamic coupling effect [32], the network adaptively calculates the importance weight $\alpha_{ij}$ of neighboring node j to target node i using a shared linear transformation matrix $W_{gat}$ and an attention mechanism, normalized via Softmax. A non-linear activation function then outputs an updated feature $h_{i}^{\left(1\right)}$ incorporating system-wide implicit coupling information. We capture the mean weight matrix $C\in R^{N\times N}$ (where $C_{ij}=\alpha_{ij}$) to serve as the topological difference feature graph.

### 2.4. Dual-Branch Decoupled Optimization Mechanism

To maintain the purity of the feature space, a spatiotemporal dual-branch two-stage decoupled optimization mechanism cuts off gradient propagation [33], isolating physical representation acquisition from topological coupling classification.

Stage One (Continuous Health Quantification): Relying on unsupervised reconstruction, latent space decoders $f_{dec,i}$ restore original inputs from features $h_{i}^{\left(0\right)}$. The mean squared reconstruction error ($L_{MSE}$) measures the physical manifold offset:

$$L_{MSE}=\frac{1}{N}\sum_{i=1}^{N}{\left|\left|h_{i}^{\left(0\right)}-{\hat{h}}_{i}^{\left(0\right)}\right|\right|}_{2}^{2}$$

Backpropagation only updates $f_{align}$ and decoders based on $L_{MSE}$. A sensitivity coefficient λ=1.0 maps the reconstruction error to a continuous state via a negative exponential function, forming the HI:

$$HI=\exp\left(-\lambda \cdot L_{MSE}\right)$$

An HI value approaching 1 indicates healthy operation; a drop below the safety threshold triggers an early warning, initiating stage two.

Stage Two (Root-Cause Diagnosis and Topology Classification): After stage one converges, network weights for $f_{align}$ are strictly frozen. The model cuts off gradient backpropagation from the classification branch to the feature extraction module, immunizing node features $h_{i}^{\left(0\right)}$ against specific fault bias. The supervised branch concatenates updated features $H^{\left(1\right)}={\left[h_{1}^{\left(1\right)},h_{2}^{\left(1\right)},\ldots ,h_{N}^{\left(1\right)}\right]}^{T}$ into an MLP classifier $f_{cls}$ to output probability prediction vector $\hat{Y}$ via cross-entropy loss. Gradients solely optimize the graph layer attention parameters and classifier weights [34], achieving task-level decoupling.

### 2.5. Anomaly Evolution Based on Topological Difference Matrix

**Figure 4:**
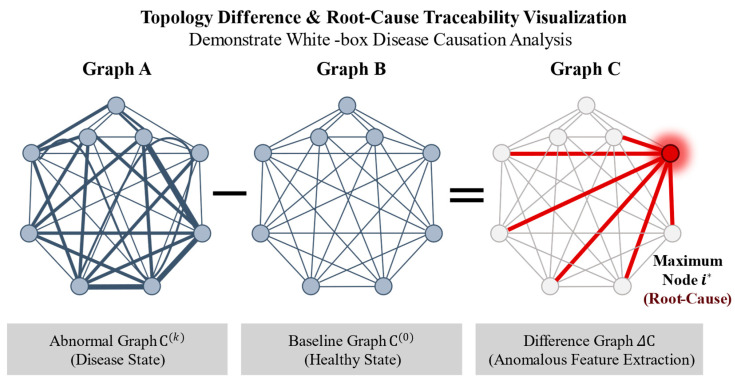
Principles of topological difference and root-cause traceability.

To endow results with physical interpretability, a topological difference inference mechanism is executed after network optimization. As illustrated in Figure 4, the underlying assumption is that specific hardware degradation locally offsets its features, triggering attention changes in neighboring nodes via implicit physical fields. Inspired by the macroscopic concept of utilizing graph topological structures for fault identification [35], we propose a novel, custom-designed topological difference inference algorithm to explicitly quantify this propagation. The core physical logic dictates that the root-cause node—acting as the “epicenter” of the anomaly—will experience the most severe distortion in its topological connection weights with surrounding subsystems. Therefore, by mathematically tracking the extreme points of the evolving weights in the graph topology, we can reversely locate the source node inducing the cascading degradation.

During inference, we extract the average attention matrix of samples classified as anomalous state k to form the anomalous coupling graph $C^{\left(k\right)}\in R^{N\times N}$. Similarly, the baseline coupling graph $C^{\left(0\right)}$ is extracted from the normal state. The differential topological attention matrix $\Delta C=\left|C^{\left(k\right)}-C^{\left(0\right)}\right|$ quantifies the absolute offset. The anomaly influence degree $I_{i}$ of node i (covering in-degree and out-degree fluctuations) is defined as

$$I_{i}=\sum_{j=1}^{N}\Delta C_{ji}+\sum_{j=1}^{N}\Delta C_{ij}.$$

Ultimately, node $i^{*}=\arg\underset{i}{\max}\left(I_{i}\right)$ is labeled as the pathogenic target inducing the system-level warning [36].

## 3. Experimental Platform and Data Preprocessing

### 3.1. Experimental Platform and Data Collection

Data were collected from industrial SLM equipment (Raycham, Nanjing City, Jiangsu Province, China) during a continuous 3-day operation, executing a massive 8582-layer printing task to fabricate a complex shoe mold. The raw material utilized was a commercial aluminum alloy (AlSi10Mg) consisting of highly spherical powder with a particle size distribution of 15–53 μm, processed under a continuous high-purity argon protective gas flow. The core process parameters were configured as follows: the optical system operated at a nominal laser power of 365 W with a focal spot diameter of 80 μm and an average scanning speed of 1180 mm/s, while the mechanical forming system deposited a constant layer thickness of 40 μm. By monitoring 7 core physical subsystems at a sampling frequency of 1 Hz, 41-dimensional heterogeneous sensory features were extracted. As shown in Table 1 and Table 2, under continuous full-load operation, a dataset of 310,220 temporal records was constructed. The dataset contains the normal state (Y=0) and 7 anomalous states originating from underlying hardware (Y∈{1, 2, …, 7}). Feature alignment mapping projects these 41 one-dimensional signals into a uniform latent space, constructing aligned input vectors for decoupled GAT computation.

### 3.2. Label Cleaning Mechanism Based on Temporal Filtering Windows

The extraction of valid degradation features from industrial SLM equipment necessitates specific data preprocessing methodologies prior to model training. During continuous full-load 3D-printing operations, hardware degradation is not an instantaneous discrete switch, but rather a continuous evolution heavily influenced by physical inertia. For instance, the degradation of the filtration system requires a temporal delay to significantly alter the chamber pressure and subsequently impact the forming quality. Consequently, directly assigning static binary fault labels (discrete step signals) to raw time-series data based solely on maintenance timestamps creates a severe temporal misalignment. This misalignment introduces label noise, such as forcibly tagging a pre-fault degradation phase as a “normal” state.

**Figure 5:**
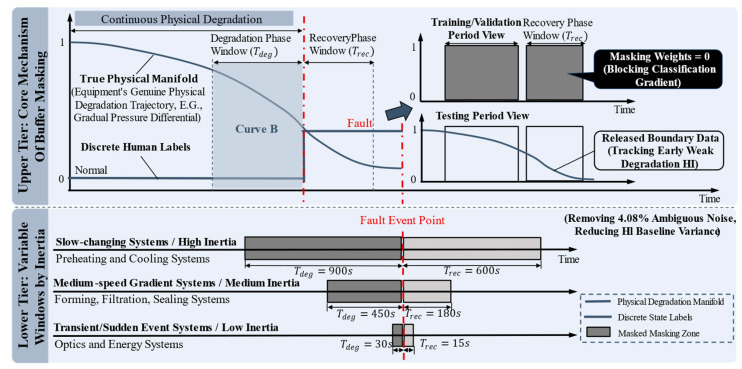
A variable transition window masking strategy based on system physical inertia.

To address the mismatch between mechanical action and physical field response, we propose a variable transition buffer masking strategy based on system physical inertia, the core mechanism of which is illustrated in Figure 5. The dataset is partitioned chronologically (70:15:15). During training and validation, the degradation window $T_{deg}$ and recovery window $T_{rec}$ are set dynamically based on system inertia, and the cross-entropy masking weight for these samples is set to 0. In the testing phase, unmasked transition data are fully released to reversely verify the model’s unsupervised HI tracking ability.

Configurations depend on system inertia:

High inertia (slow-changing): Preheating and cooling systems ($T_{deg}=900\;s$, $T_{rec}=600\;s$).

Medium inertia (gradual): Forming, filtration, and sealing systems ($T_{deg}=450\;s$, $T_{rec}=180\;s$).

Low inertia (transient): Optics and energy systems ($T_{deg}=30\;s$, $T_{rec}=15\;s$).

This strategy identified 22 state switches, masking 4.08% of ambiguous data. It successfully isolated boundary label noise, reducing HI baseline variance while retaining over 95% of unambiguous data.

### 3.3. Evaluation Metrics

To implement two-stage decoupled optimization, joint loss backpropagation is completely severed. The sliding window size is W=32. The Pearson correlation threshold for the normal baseline is τ=0.1. The model is built on a 24 GB VRAM platform, utilizing the Adam optimizer (learning rate 0.001). To address severe industrial data imbalance, accuracy (ACC) is strictly defined as the single-class correct prediction ratio over actual total class samples (functioning identically to Detection Rate). Using stage one’s unsupervised HI to filter global false alarms, single-class ACC and global Macro-F1 scores constitute the core evaluation benchmarks.

## 4. Results and Discussion

### 4.1. Overall Diagnostic Performance Analysis

To verify effectiveness, we evaluated four data-driven models under identical setups: 1D-CNN and LSTM (representing baseline sequence architectures assuming independent channels), Static GCN (representing static physical mapping via fixed Pearson adjacency matrices), and an End-to-End VAE-MLP (the core control group possessing identical parameters but employing joint end-to-end backpropagation). The experimental results are shown in Table 3.

**Figure 6:**
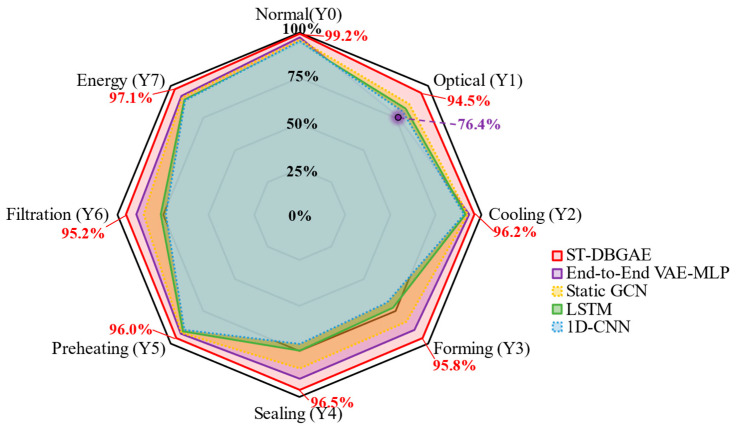
Radar chart comparing single-class accuracy (ACC) across subsystems of multiple models.

**Figure 7:**
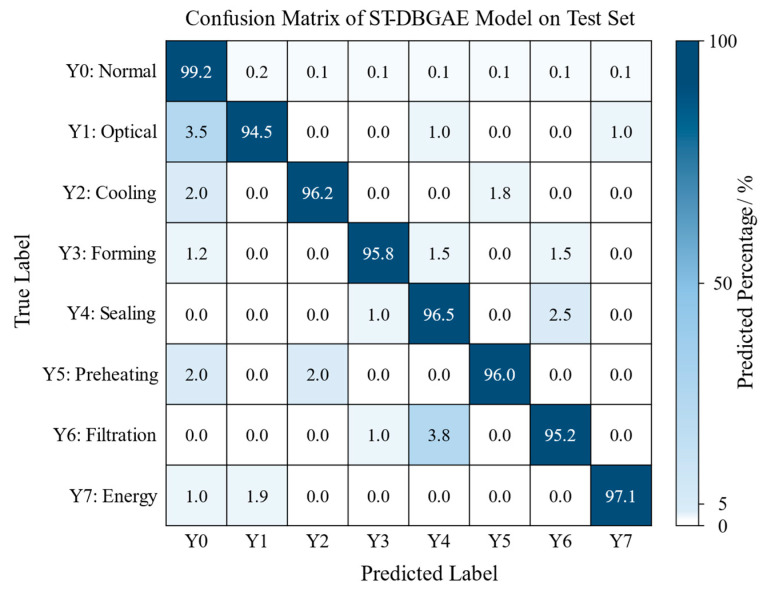
ST-DBGAE model confusion matrix heatmap.

Furthermore, the radar chart comparing single-class accuracy and the confusion matrix of the ST-DBGAE model are presented in Figure 6 and Figure 7 respectively. Analysis yields three primary conclusions. First, explicit spatiotemporal topological modeling is a prerequisite for deconstructing cross-system cascading degradation. The feature concatenation mechanism fails drastically against pneumatic coupling circuits (Y4,Y6). When filter clogging (Y6) triggers main circuit wind speed drops causing chamber pressure anomalies (Y4), 1D-CNN recognition rates plummet to 71.2% and 73.5%. The static graph structure improves these to 84.5%, demonstrating the necessity of explicit mapping. Second, dynamic sparse topologies outperform static physical graphs. Thermal accumulation dynamically alters implicit physical coupling. Static GCN’s solidified matrices trigger over-smoothing, whereas ST-DBGAE achieves 96.5% overall accuracy by adaptively adjusting coupling link weights. Third, the two-stage decoupled mechanism successfully mitigates feature submersion. The end-to-end model suffers significantly on weak signals (e.g., optical system Y1), dropping to 76.4% ACC because large-variance macroscopic anomalies interfere via loss gradients. ST-DBGAE blocks downward interference, surging Y1 accuracy to 94.5%.

### 4.2. Ablation Study and Decoupling Mechanism Validation

**Figure 8:**
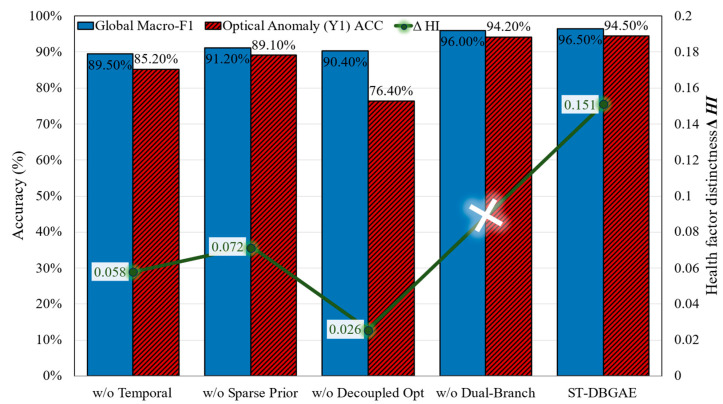
Combined performance degradation chart for core mechanism ablation experiments.

Four variant models were constructed under the W=32 benchmark to verify key independent mechanisms. The results of the ablation experiments are presented in Table 4, while the performance degradations of each variant model are visualized and compared in Figure 8.

In addition to the global Macro-F1 score, the single-class accuracy of the optical anomaly (Y=1) is specifically isolated as a core evaluation metric in this ablation study. The physical rationale is that the optical subsystem’s signals (e.g., galvanometer voltage and temperature drift) possess extremely small amplitude variations, making them the most vulnerable to the “feature submersion” effect caused by the large-variance signal oscillations of macroscopic pneumatic or mechanical systems. If the internal decoupling mechanisms fail, the severe degradation of this specific weak node might be mathematically masked by the stable high accuracy of other robust nodes when only calculating the average Macro-F1 score. Therefore, tracking the performance fluctuation of the optical subsystem serves as the most stringent “stress test” to validate the necessity of the proposed decoupled isolation modules. The data confirms the algorithmic significance of core components. Removing the temporal alignment layer (Macro-F1 drops to 89.5%) proves that electromechanical failure accumulates over time.

Employing a complete graph (w/o Sparse Prior) forces non-interacting subsystems to connect, causing over-smoothing and dropping the normal HI mean to 0.763. Restoring end-to-end training (w/o Decoupled Opt) obliterates HI distinctness (Δ HI drops to 0.026) and optical accuracy (76.4%), confirming that classification gradient backflow irrevocably corrupts weak node independence. Stripping the dual-branch removes the ability to continuously quantify degradation.

**Figure 9:**
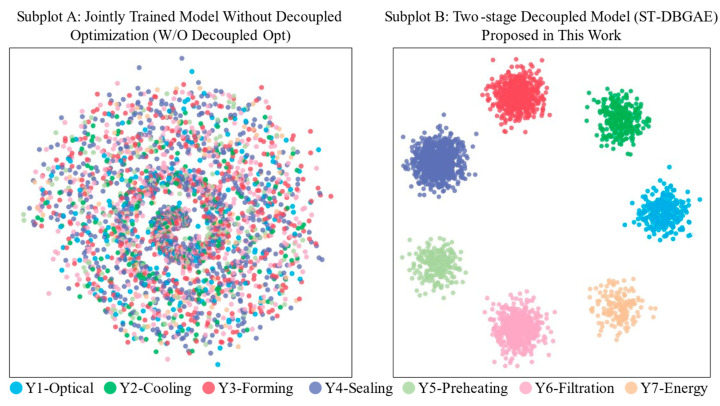
Underlying node features in the latent space before and after decoupling.

Furthermore, to visually demonstrate the internal mechanism of the proposed decoupling strategy, t-Distributed Stochastic Neighbor Embedding (t-SNE) was employed to project the high-dimensional latent node features into a 2D space, as illustrated in Figure 9. Subplot A displays the feature distribution of the baseline model trained without decoupled optimization. Driven by the joint end-to-end classification loss, the features of different physical subsystems are severely entangled, forming a spiral where distinct clusters permeate each other and lose their physical boundaries. In contrast, Subplot B displays the latent space of our proposed ST-DBGAE after the stage one decoupled feature alignment. The underlying node features exhibit a distribution with high intra-class cohesion and low inter-class coupling. This clear separation explicitly confirms that the two-stage isolation strategy successfully shields the underlying hardware features from global gradient cross-interference, thereby laying a pure, physically bounded foundation for the subsequent dynamic topology inference.

### 4.3. Health Evolution Tracking and Root-Cause Traceability Analysis

#### 4.3.1. Full-Lifecycle HI Degradation Trajectory Assessment

Traditional discrete networks struggle to characterize continuous decline. ST-DBGAE’s unsupervised branch outputs a continuous HI scalar via $L_{MSE}$. In normal operating conditions (Y=0), test sample sequences densely populate the high baseline band (mean 0.791), suppressing false-alarm background noise.

**Figure 10:**
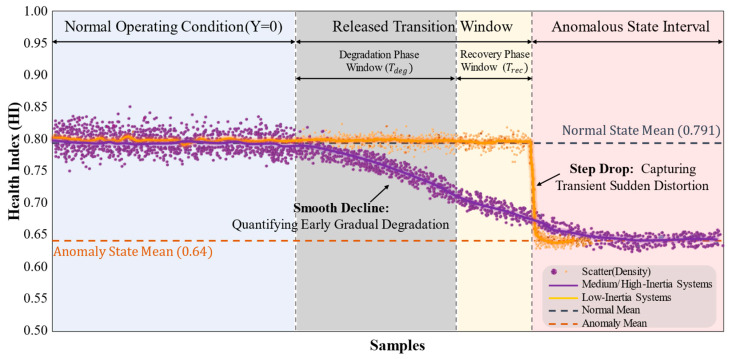
Multimodal degradation trajectory map of the full-lifecycle health index (HI).

As clearly depicted in the multimodal degradation trajectory map (Figure 10), during the completely released “transition window,” the HI traces a continuous evolution trajectory highly consistent with physical reality, dropping to an anomalous mean of 0.640. For high/medium-inertia systems (filtration Y=6, preheating Y=5), the curve exhibits a smooth, gradual decline, perfectly quantifying time-integrated quantitative changes like filter pore clogging. For low-inertia systems (optical Y=1, energy Y=7), the curve accurately reflects sudden step drops.

#### 4.3.2. Dynamic Topological Difference Inference

To verify the analytical capability of the stage two attention aggregation mechanism for implicit cross-system cascading degradation, and to strictly rule out the possibility of random coincidences, we extracted sample groups judged as anomalous across different subsystems. It is important to emphasize that these extracted groups encompass multiple independent degradation instances that occurred throughout the continuous 3-day, 8582-layer printing operation. For each anomalous state k, the average coupling graph $C^{\left(k\right)}$ across all instances was subtracted from the normal baseline graph $C^{\left(0\right)}$ to derive the statistically significant difference topology matrix ΔC.

**Figure 11:**
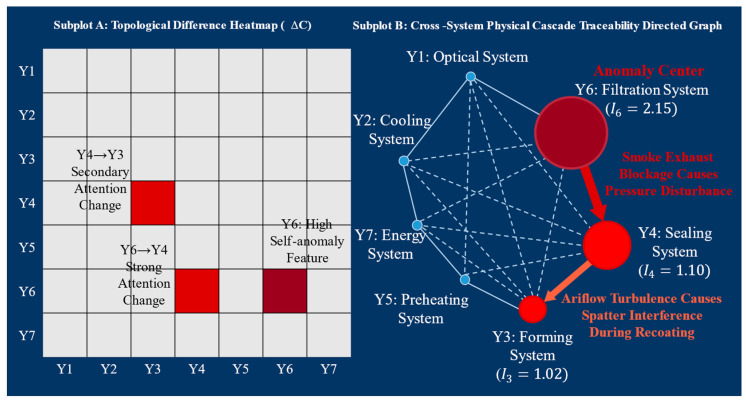
Dynamic topology difference heatmap and cross-system cascading traceability graph.

To verify traceability, the sample group judged as a filtration system anomaly (Y=6) is extracted. As shown in the difference graph in Figure 11, the fluctuation extremes anchor precisely at the filtration system node, presenting directed edges pointing to the sealing and forming systems. This maps perfectly to physical mechanisms: coarse filter element clogging reduces smoke exhaust capacity; blocked exhaust disrupts sealing system airflow (chamber pressure disturbance); the resulting turbulence fails to clear spatter; and cooled spatter protrusions cause recoater blade collisions (forming torque anomaly). This confirms the model reliably captures directed propagation links crossing physical boundaries.

**Figure 12:**
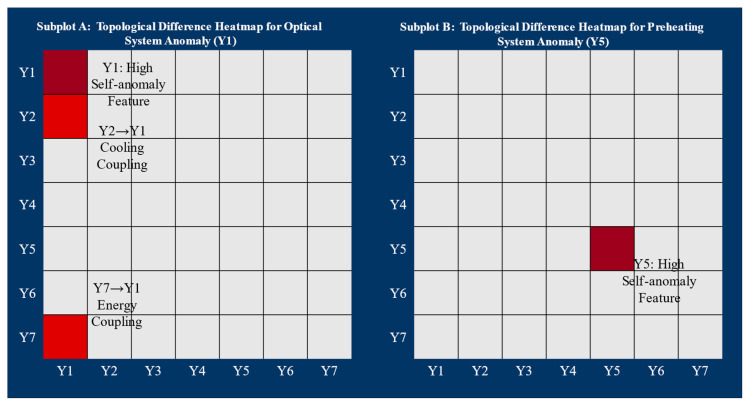
Dynamic topology difference heatmaps for direct thermo-electric coupling (**A**) and independent thermal inertia (**B**).

To further demonstrate the robust causality of our approach across varying physical fields and to verify its immunity to spurious correlations, we additionally analyzed the topological difference matrices for the optical system (Y=1) and the preheating system (Y=5), as visualized in Figure 12.

Direct Thermo-Electric Coupling (Optical System Anomaly, Y=1): As shown in Figure 12A, when an optical system anomaly occurs, the attention weight shifts directly point toward the cooling system (Y=2) and the energy system (Y=7). The physical causality is explicitly mapped: the optical galvanometer’s temperature and working stability are heavily dependent on the high- and low-temperature water flow from the cooling system, while the galvanometer’s voltage load is structurally coupled with the laser power output from the energy system. The model successfully quantifies this strong, direct thermo-electric hardware dependency.

Independent Thermal Inertia (Preheating System Anomaly, Y=5): To verify whether the model suffers from spurious correlations—such as falsely linking a preheating anomaly to an optical degradation due to generalized environmental heat soaking—we analyzed the preheating system anomaly. Figure 12B reveals that the preheating system acts as a highly independent node with negligible cross-system attention shifts. The optical system’s attention weights remain entirely undisturbed. This proves that the stage one decoupled feature alignment strictly preserves the physical independence of the components, while the sparse prior threshold τ effectively filters out generalized background thermal crosstalk, guaranteeing the extraction of true physical causality.

### 4.4. Systematic Evaluation of Model Boundaries and Robustness

#### 4.4.1. Generalization Assessment Against Extreme Signal Drift and Noise

SLM long-term operations inevitably involve sensor aging (e.g., thermocouple drift) and background noise. Two extreme test scenarios were implemented on the test set: (1) a ±15% baseline drift on environmental sensing features; (2) mild (SNR = 20 dB) and strong (SNR = 15 dB) noise injection to systematically evaluate the model’s robustness against random perturbations on the factory floor.

**Figure 13:**
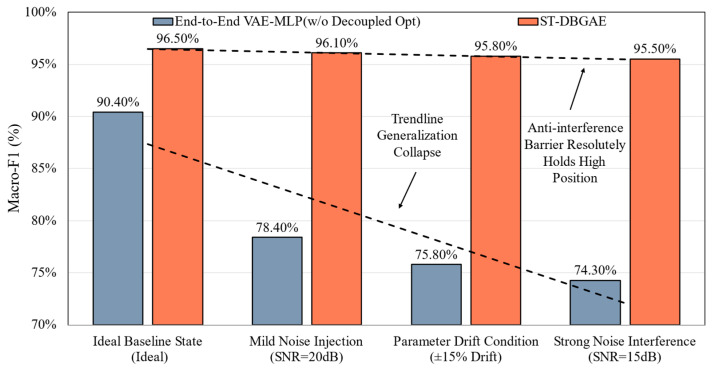
Comparison of generalization capabilities across operating conditions and in high-noise environments (comparing the proposed ST-DBGAE with the baseline model without decoupling).

As shown in Figure 13, results indicate that the performance of the end-to-end baseline model (i.e., the jointly trained VAE-MLP model without the proposed decoupled optimization) decays significantly, with the Macro-F1 score dropping below 75.8%. This indicates its high dependence on numerical fitting of specific distributions. Conversely, ST-DBGAE maintains robust cross-condition performance (Macro-F1 90.4%). This robustness against random perturbations is primarily attributed to the inherent low-pass filtering property of the graph attention aggregation mechanism, which effectively smooths out high-frequency random sensor fluctuations before topological causality reasoning. Feature alignment effectively strips numerical dimensional dependence, and topology inference focuses entirely on relative attention shifts, shielding the architecture from environmental disturbances.

#### 4.4.2. Sensitivity of Sparse Prior Threshold τ

**Figure 14:**
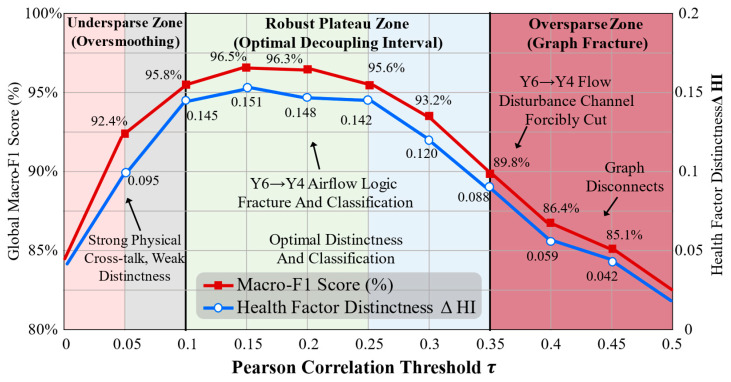
Multi-metric sensitivity and connectivity boundary test plots for the sparsity prior threshold τ.

The Pearson correlation threshold τ determines topology connectivity and serves as a highly critical calibration parameter that establishes the physical boundary for cross-system interactions. An optimal calibration of τ is essential to balance the retention of weak causal signals against the filtration of redundant topological crosstalk. By sampling τ equidistantly at 0.05 steps within $\left[0.0,\;0.5\right]$, we systematically evaluated this calibration process. As shown in Figure 14, the testing performance follows an inverted U-shaped curve.

When τ< 0.05, excessive noise edges cause over-smoothing. When $\tau \in \left[0.10,\;0.25\right]$, the model cuts off redundant noise while retaining real interaction channels (Macro-F1 > 95.5%). When τ> 0.30, real cross-system edges are forcibly cut, interrupting traceability links and plummeting recognition rates. The empirical calibration determines τ=0.15 as the optimal configuration, where the model achieves peak diagnostic accuracy. This stable performance plateau demonstrates excellent engineering deployment feasibility.

## 5. Conclusions and Future Work

### 5.1. Conclusions

Addressing the challenges of strong multi-physical subsystem coupling and cascading fault evolution in SLM equipment under continuous operation, this paper proposes a condition monitoring and anomaly traceability method based on a spatiotemporal dual-branch decoupled graph autoencoder (ST-DBGAE). The research yields three core outcomes:

1. Local Spatiotemporal Feature Alignment: By projecting multi-source signals into a unified latent space via parallel local mappings, the method effectively overcomes the feature submersion problem inherent in global concatenation, strictly maintaining the feature independence of distinct physical subsystems.
2. Dynamic GAT with Sparse Priors: Utilizing mutual information under normal conditions to establish initial boundaries, the attention mechanism adaptively quantifies dynamic weights triggered by implicit thermal/fluid interactions, averting the failure of static adjacency matrices under complex time-varying conditions.
3. Two-Stage Decoupled Optimization and Topological Difference Inference: By cutting off cross-interference from global classification backpropagation, continuous HI quantification is realized. Reversely comparing the dynamic topological difference matrix before and after anomalies accurately anchors the root-cause hardware and its propagation paths.

Validation on real industrial equipment yields a comprehensive Macro-F1 score of 96.5% across eight operating states. High robustness is maintained under condition drift and strong background noise, providing a condition monitoring solution that balances high accuracy and strict physical interpretability.

### 5.2. Limitations and Future Work

Despite effective validation, the current research exhibits limitations regarding the deep-seated needs of full-lifecycle management. The architecture currently prioritizes real-time monitoring and reverse traceability; a forward-looking time-series evolution extrapolation mechanism is yet to be established for direct remaining useful life (RUL) prediction. Furthermore, the reliance on a predefined static correlation threshold limits adaptive evolution capabilities when facing entirely new forming materials or generational process parameter shifts.

Future research will extend towards the dynamic prediction (prognostics) of multisystem coupling situations. First, we will explore spatiotemporal predictive GNN architectures, integrating graph recurrent networks into the decoupled representation to predict joint manifolds and topological connection weights across future time steps. Second, we will conduct physics-and-data dual-driven cascading failure prediction, embedding thermal radiation and fluid dynamics boundary constraints into graph node inference. This will enable the model to forward-infer cascading spread paths the moment extremely weak early degradation signals are captured, developing the health management closed loop from passive traceability to active prediction.

## Figures and Tables

**Table 1:** The feature distribution across the SLM subsystems.

Subsystem	Component	Key Parameters (Variables)
Optical	Galvanometer	Voltage, Temperature, Humidity, Head Temperature
Cooling	High-Temperature Water	Temperature, Flow Rate
	Low-Temperature Water	Temperature, Flow Rate
Forming	Build Cylinder	Current Position, Current Velocity, Right-Side Position, Torque
	Recoater Blade	Current Position, Current Velocity
	Powder Delivery	Feed Multiplier, Weight
Sealing	Processing Chamber	Pressure, Oxygen Concentrations 1 and 2
	Main Circuit	Inlet Air Velocity, Pressure, Temperature Return Air Velocity, Pressure, Temperature
	Branch Circuit	Return Air Velocity, Pressure, Temperature
Preheating	Substrate Plate	Temperature (Zones 1, 2, 3)
Filtration	Pressure	Total Inlet Air, Backblow Air, Compressed Air
	Inlet Air Flow	Rear Panel, Main Chamber, Protective Window
	Differential Pressure	Coarse Filters 1 and 2
Energy	Lasers	Actual Power (Lasers 1, 2)

**Table 2:** Data distribution in subsystems.

Dataset	Normal (Y = 0)	Optical (Y = 1)	Cooling (Y = 2)	Forming (Y = 3)	Sealing (Y = 4)	Preheating (Y = 5)	Filtration (Y = 6)	Energy (Y = 7)
Quantity	290,220	1960	1960	3900	5840	1460	3900	980

**Table 3:** Classification performance of diagnostic models.

Diagnostic Model	Y0	(Y1)	(Y2)	(Y3)	(Y4)	(Y5)	(Y6)	(Y7)	Macro-F1
1D-CNN	95.0	80.1	90.5	68.4	71.2	89.4	73.5	88.7	81.2
LSTM	94.5	82.3	91.2	72.5	74.8	90.6	76.1	89.3	83.5
Static GCN	96.2	85.6	92.4	83.2	84.5	91.8	85.4	91.1	88.1
End-to-End VAE-MLP	97.1	76.4	93.1	89.5	90.2	92.5	89.7	91.8	90.4
ST-DBGAE	99.2	94.5	96.2	95.8	96.5	96.0	95.2	97.1	96.5

**Table 4:** Ablation study results.

Model Variants	Optics (Y1) ACC	Macro-F1	Normal HI Mean	Abnormal HI Mean	Δ HI	Ablation Description
w/o Temporal	85.2%	89.5%	0.812	0.754	0.058	Replaced LSTM with MLP
w/o Sparse Prior	89.1%	91.2%	0.763	0.691	0.072	Set τ = 0 (complete graph)
w/o Decoupled Opt	76.4%	90.4%	0.741	0.715	0.026	Restored end-to-end training
w/o Dual-Branch	94.2%	96.0%	N/A	N/A	N/A	Removed unsupervised branch
ST-DBGAE	94.5%	96.5%	0.791	0.640	0.151	Fused advantages

## Data Availability

Data will be made available upon request.

## References

[1] Yap C.Y., Chua C.K., Dong Z.L., Liu Z.H., Zhang D.Q., Loh L.E., Sing S.L. (2015). Review of selective laser melting: Materials and applications. Appl. Phys. Rev..

[2] Fang Q., Xiong G., Zhou M.C., Tamir T.S., Yan C.B., Wu H., Shen Z., Wang F.Y. (2022). Process monitoring, diagnosis and control of additive manufacturing. IEEE Trans. Autom. Sci. Eng..

[3] Uhlmann E., Pontes R.P., Geisert C., Hohwieler E. (2018). Cluster identification of sensor data for predictive maintenance in a selective laser melting machine tool. Procedia Manuf..

[4] Chen L., Moon S.K. (2024). In-situ defect detection in laser-directed energy deposition with machine learning and multi-sensor fusion. J. Mech. Sci. Technol..

[5] Petrich J., Snow Z., Corbin D., Reutzel E.W. (2021). Multi-modal sensor fusion with machine learning for data-driven process monitoring for additive manufacturing. Addit. Manuf..

[6] Gurav V., Upadhyay A., Sakhare H. (2025). An explainable lightweight framework for process control and fault detection in additive manufacturing. J. Manuf. Mater. Process..

[7] Xie G., Walker J., Snow Z., Diehl B.G., Reutzel E.W. (2025). Deep learning-based multisensor fusion for in situ defect prediction in additive manufacturing. Proc. SPIE.

[8] Simsir U. (2026). Multi-sensor process monitoring and fault diagnosis for multi-mode industrial servomotor systems with fault classification and RUL prediction: A representative case study for smart manufacturing applications. Processes.

[9] Bevans B., Barrett C., Spears T., Gaikwad A., Riensche A., Halliday H.S., Rao P. (2023). Heterogeneous sensor data fusion for multiscale, shape agnostic flaw detection in laser powder bed fusion additive manufacturing. Virtual Phys. Prototyp..

[10] Sindhu S., Nagpal A., Kumar A., Namrata N. (2024). Intelligent fault diagnosis of surface level defects in stainless steel plates through fused sensor data. AIP Conf. Proc..

[11] Zhang Z., Wei C., Xie S., Zhang W., Wen L. (2024). A new multisensor feature fusion KAN network for autonomous underwater vehicle fault diagnosis. IEEE Trans. Instrum. Meas..

[12] Mojumder S., Halder P., Tonge T. (2026). Multimodal learning of melt pool dynamics in laser powder bed fusion. Prog. Addit. Manuf..

[13] Luo Q., Huang N., Bartles D.L., Beese A.M. (2025). Geometry-informed multimodal variational autoencoder for real-time prediction of properties for Ti–6Al–4V fabricated using PBF-LB. J. Intell. Manuf..

[14] Nie Q., Geng J., Liu C. (2026). A review of fault diagnosis methods: From traditional machine learning to large language model fusion paradigm. Sensors.

[15] Wang J. (2025). Development of an online defect detection system for additive manufacturing based on multi-sensor fusion. ITM Web Conf..

[16] Gao Y., Zhong Z., Ma M., Zhang Z., Zhang Y., Wang C. (2024). Physics-embedded recurrent graph neural network for fault diagnosis of complex systems. IEEE Access.

[17] Liu R., Zhang Q., Lin D., Zhang W., Ding S.X. (2024). Causal intervention graph neural network for fault diagnosis of complex industrial processes. Reliab. Eng. Syst. Saf..

[18] Xing K., Wang Y., Lu H., Yun D. Research on fault diagnosis and predictive maintenance algorithm of complex industrial system based on graph neural network. Proceedings of the 2025 Asia Conference on Energy Conversion Systems and Power Electronics (AECSPE).

[19] Ma J., Huang J., Liu S., Luo J., Jing L. (2024). A self-attention Legendre graph convolution network for rotating machinery fault diagnosis. Sensors.

[20] Jiang L., Wang S., Zhang T., Zhang X. (2024). Semi-supervised few-shot fault diagnosis driven by multi-head dynamic graph attention network under speed fluctuations. Digit. Signal Process..

[21] Wen W., Qin J., Chang Q. (2026). Semi-supervised graph attention network for screw pump fault diagnosis: Revealing the dynamic coupling of multi-source information. Entropy.

[22] Wang B., Zhao S. (2025). MTAGCN: Multi-task graph-guided convolutional network with attention mechanism for intelligent fault diagnosis of rotating machinery. Machines.

[23] Xiao J., Lan B., Jiang C., Terzi S., Zheng C., Eynard B., Anwer N., Huang H. (2025). Graph attention-based knowledge reasoning for mechanical performance prediction of L-PBF printing parts. Int. J. Adv. Manuf. Technol..

[24] Liu Q., Liu W., Bian H., Xing F. (2026). Deep spatiotemporal condition monitoring and subsystem fault classification for selective laser melting equipment. Coatings.

[25] Yu H., Khan F., Garaniya V. (2015). Modified independent component analysis and Bayesian network-based two-stage fault diagnosis of process operations. Ind. Eng. Chem. Res..

[26] Duan H., Chen G., Yu Y., Du C., Bao Z., Ma D. (2025). DyGAT-FTNet: A dynamic graph attention network for multi-sensor fault diagnosis and time–frequency data fusion. Sensors.

[27] Li C., Ma Z., Zeng Y., Yang Z., Li J., Yang Z., Xiong J., Niu S., Wang Z., Zhao H. (2026). Machine learning-driven alignment architecture of heterogeneous data with transient varying semantics. Nat. Commun..

[28] Zhang W., Liu Z., Jia Z., Wang X., Yan W., Wang K. (2025). Application of a multimodal deep learning model based on recursive fusion feature map with transformer-TCN for complex fault diagnosis of flying wing UAV actuators. IEEE Trans. Instrum. Meas..

[29] Yu J., Huang J., Fu X., Jin Y., Yan X., Yang X. (2025). Disentangled feature representation based on multiscale content learning in industrial heterogeneous nonstationary fault detection. IEEE Trans. Instrum. Meas..

[30] Aryal B., Modekwe G., Lu Q. (2026). Multi-level temporal graph networks with local-global fusion for industrial fault diagnosis. arXiv.

[31] Han Y., Liu X., Guo C., Wu H., Liu M., Geng Z. (2024). Improved Pearson correlation coefficient-based graph neural network for dynamic soft sensor of polypropylene industries. Ind. Eng. Chem. Res..

[32] Yang C., Liu J., Hu Y., Wu B., Shi T. (2024). Dynamic graph-driven rotating machine fault diagnosis: An adaptively updating cross-domain relationship information. IEEE Trans. Ind. Inform..

[33] Chen C., Shi J., Feng L., Yi H., Wang C. (2024). A two-stage fault diagnosis method with rough and fine classifiers for phased array radar transceivers. IEEE Trans. Instrum. Meas..

[34] Komorska I., Puchalski A. (2024). Condition monitoring using a latent space of variational autoencoder trained only on a healthy machine. Sensors.

[35] Zhao S., Zhang H., Sun B., Wang Y. (2026). Spatio-temporal graph neural networks for anomaly detection in complex industrial processes. Sensors.

[36] Shan X. (2025). Fault Identification and Localization in Distribution Grids Based on an Attention-Hybrid Graph Neural Network. Ph.D. Dissertation.

